# Diagnostic performance of smear microscopy and incremental yield of Xpert in detection of pulmonary tuberculosis in Rwanda

**DOI:** 10.1186/s12879-016-2009-x

**Published:** 2016-11-08

**Authors:** Jean Claude Semuto Ngabonziza, Willy Ssengooba, Florence Mutua, Gabriela Torrea, Augustin Dushime, Michel Gasana, Emmanuel Andre, Schifra Uwamungu, Alaine Umubyeyi Nyaruhirira, Dufton Mwaengo, Claude Mambo Muvunyi

**Affiliations:** 1 National Reference Laboratory Division, Rwanda Biomedical Centre, Kigali, Rwanda; 2 Department of Medical Microbiology, Makerere University College of Health Sciences, School of Biomedical Sciences, Kampala, Uganda; 3 Department of Global Health and Amsterdam Institute of Global Health and Development, Academic Medical Center, University of Amsterdam, Amsterdam, Netherlands; 4 Department of Medical Microbiology, School of Medicine, College of Health Sciences, University of Nairobi, Nairobi, Kenya; 5 Mycobacteriology Unit, Institute of Tropical Medicine Prince Leopold, Antwerp, Belgium; 6 Tuberculosis and other respiratory diseases Division, Rwanda Biomedical Centre, Kigali, Rwanda; 7 Pôle de microbiologie médicale, Institut de Recherche Expérimentale et Clinique, Université catholique de Louvain, Brussels, Belgium; 8 Biomedical Laboratory Sciences Department, School of Allied Health Sciences, College of Medicine and Health Sciences, University of Rwanda, Kigali, Rwanda; 9 Management Sciences for Health, Pretoria, South Africa; 10 Clinical Biology Department, School of Medicine and Pharmacy, College of Medicine and Health Sciences, University of Rwanda, Butare, Rwanda

**Keywords:** Tuberculosis microscopy in routine conditions, LED-FM versus ZN, Incremental yield of Xpert, Tuberculosis diagnosis in Rwanda

## Abstract

**Background:**

Tuberculosis control program of Rwanda is currently phasing in light emitting diode-fluorescent microscopy (LED-FM) as an alternative to Ziehl-Neelsen (ZN) smear microscopy. This, alongside the newly introduced Xpert (Cepheid, Sunnyvale, CA, USA) is expected to improve diagnosis of tuberculosis and detection of rifampicin resistance in patients at health facilities. We assessed the accuracy of smear microscopy and the incremental sensitivity of Xpert at tuberculosis laboratories in Rwanda.

**Methods:**

This was a cross-sectional study involving four laboratories performing ZN and four laboratories performing LED-FM microscopy. The laboratories include four intermediate (ILs) and four peripheral (PLs) laboratories. After smear microscopy, the left-over of samples, of a single early-morning sputum from 648 participants, were tested using Xpert and mycobacterial culture as a reference standard. Sensitivity of each test was compared and the incremental sensitivity of Xpert after a negative smear was assessed.

**Results:**

A total of 96 presumptive pulmonary tuberculosis participants were culture positive for *M. tuberculosis*. The overall sensitivity in PL of ZN was 55.1 % (40.2–69.3 %), LED-FM was 37 % (19.4–57.6 %) and Xpert was 77.6 % (66.6–86.4 %) whereas in ILs the same value for ZN was 58.3 % (27.7–84.8 %), LED-FM was 62.5 % (24.5–91.5 %) and Xpert was 90 (68.3–98.8 %). The sensitivity for all tests was significantly higher among HIV-negative individuals (all test *p* <0.05). The overall incremental sensitivity of Xpert over smear microscopy was 32.3 %; *p* < 0.0001. The incremental sensitivity of Xpert was statistically significant for both smear methods at PL (32.9 %; *p* = 0.001) but not at the ILs (30 %; *p* = 0.125) for both smear methods.

**Conclusions:**

Our study findings of the early implementation of the LED-FM did not reveal significant increment in sensitivity compared to the method being phased out (ZN). This study showed a significant incremental sensitivity for Xpert from both smear methods at peripheral centers where majority of TB patients are diagnosed. Overall our findings support the recommendation for Xpert as an initial diagnostic test in adults and children presumed to have TB.

## Background

Despite the recommendations of the World Health Organization to use Xpert [1] as a first line diagnostic test, smear microscopy remains the most available and affordable test in low-income countries. Microscopy is inexpensive and highly specific in areas where there is a high prevalence of tuberculosis. However, it has several limitations including the fact that it is examiner-, technique-, and prevalence-dependent and in addition, it lacks sensitivity [2].

Studies evaluating the performance of LED-FM have shown that in addition to higher sensitivity (an average of 10 % higher than conventional ZN), it had qualitative, operational and cost advantages over both the conventional FM and ZN. On the basis of these findings, the World Health Organization (WHO) recommended in 2011 to replace conventional FM with LED-FM and phase in LED-FM as an alternative to ZN microscopy [3]. On the other hand, in 2010 WHO recommended that Xpert be used at district and sub-district levels as the initial diagnostic test in individuals suspected of having MDR-TB or HIV-associated tuberculosis. The WHO further updated recommendations on the use of Xpert including a follow-on test for smear-negative patients in other settings [1]. In a Cochrane meta-analysis, sensitivity and specificity of Xpert compared with culture were 88 % (95 % CI 83 to 92 %) and 98 % (97 to 99 %), among smear-positive cases, and 98 % (97 to 99 %) and 68 % (60 to 75 %) among smear-negative cases [4].

In Rwanda, the national tuberculosis control program has started the phase-in of LED-FM as an alternative to ZN microscopy in peripheral (PLs) and intermediate health facility laboratories (ILs). To date, 40 % of laboratories have implemented LED-FM and 16 health facilities started using Xpert for TB detection in 2012. A sample transportation system was organized to facilitate transfer of samples from health facilities without an Xpert machine. The ILs or district hospital laboratories have an average of six qualified laboratory technologists and they are supervised, trained and mentored by the National Reference Laboratory (NRL) whereas peripheral laboratories or health center laboratories have an average of two laboratory technicians and they are subsequently supervised, trained and mentored by ILs. In regards to the workload, PLs test more than 75 % of pulmonary tuberculosis presumptive yet most of these facilities have very few technologists who also perform other requested laboratory tests. Based on the countrywide data of external quality assurance of smear microscopy (annual blind rechecking and proficiency panel test), the results of PLs tend to be better than those of ILs. However, no study of accuracy has been done to assess the significance of this difference. Therefore, this study aimed to determine the sensitivity of smear microscopy and the incremental gain of Xpert for the detection of pulmonary tuberculosis at PLs and ILs to support the scale up of this new molecular technology. The present study assessed the performance of the two sputum smear microscopy techniques and the incremental yield of Xpert over microscopy among individuals with presumptive pulmonary tuberculosis, taking mycobacterial culture as the reference standard.

## Methods

### Setting, study design and population

This was a cross-sectional study involving eight health facilities which were purposively selected due to the high numbers of presumptive pulmonary tuberculosis recorded in the year 2013. Four PLs (two performing ZN and two performing LED-FM) and four intermediate laboratories (two performing ZN and two preforming LED-FM). Based on quality control of smear microscopy (QC) data of 2012 and 2013 these eight laboratories performed equally well, though the QC for intermediate is performed by the NRL whereas QC for peripheral are subsequently done by ILs.

After smear microscopy, the left-over of the samples, of a single early-morning sputum from 648 new presumptive pulmonary tuberculosis patients, were tested using Xpert and mycobacterial culture as a reference standard.

### Laboratory procedures

For each eligible participant, three to five mL of morning sputum specimen were collected in a clean plastic container with wide-mouthed, screw-capped and leak proof. A direct sputum smear was prepared, stained and examined by laboratory technicians at health facility laboratory. The left-over of sputum specimens and the examined corresponding sputum smear were immediately shipped to the tuberculosis laboratory of NRL. Sputum specimens not shipped immediately were refrigerated (4 to 8 °C). All sputum specimens collected were transported in a cool box (4–8 °C) and were processed on the same day at NRL TB laboratory.

At the NRL, sputum specimens were recorded and decontaminated using N-Acetyl-L-Cysteine Sodium hydroxide (NALC-NaOH) procedure followed by neutralization with phosphate buffer, centrifuged and the deposits (0.5 ml) inoculated in Mycobacterial growth indicator tube (BBL MGIT, Becton and Dickson, Franklin Lakes, NJ USA) and two Home-made Lowenstein Jensen (LJ) tubes respectively. The remaining pellet was used to prepare a smear and to run Xpert. For Xpert 0.5 mL of decontaminated and concentrated sputum was added to 1.5 mL of the sample reagent (i.e., a ratio of 1:3). After 15 min, two mL of the mixture was added to the Xpert cartridge and then run in the machine in accordance with manufacturer’s guide (Cepheid, Sunnyvale, CA, USA). The smears prepared from pellet were stained using auramine for LED-FM examination and NRL results were considered final for those who tested negative at the health facility. Inoculated MGIT were incubated in an automated BD BACTEC 960 machine for up to 42 days according to manufacturer’s guide (MGIT, Becton and Dickson, Franklin Lakes, NJ USA) while the two LJ tubes were incubated in manual incubator at 37 °C and inspected weekly for up to eight weeks. Positive cultures were confirmed for presence of acid fast bacilli by ZN microscopy and strain identification was done using an immunochromatographic test (SD MPT64TB Ag kit; SD Bioline, South Korea). Reexamination of smear from health facility at NRL, results of concentrated smear and Xpert provided preliminary results for treatment of pulmonary tuberculosis cases missed by health facility laboratories. The final results were provided by Mycobacterial culture.

### Data management and analysis

As the presence of MTB cannnot be excluded among contaminated cultures and cultures positive for non tuberculous mycobacterial (NTM), these results were excluded from the analysis as they may have led to an under-estimation of the sensitivity of Xpert or smear microscopy. The sensitivity and specificity was calculated for each method and type of health facility stratified by HIV-status using MGIT and/or LJ culture as gold standard. The incremental sensitivity of Xpert test to smear microscopy method was defined as the percentage of smear microscopy negative but Xpert positive by health facility among culture positive for *M. tuberculosis.* The McNemar statistical test was used to assess the significance of the differences in results obtained from smear microscopy using ZN versus LED-FM and the incremental sensitivity of Xpert. Based on these results, we compared the effectiveness of diagnostic strategies to propose the most accurate algorithm for the diagnosis of pulmonary tuberculosis at PLs and ILs. A *p*-value <0.05 was considered statistically significant. All data analysis was performed using SPSS version 21.0 software (Armonk, NY: IBM Corp.).

## Results

### Participants’ characteristics and microbiological profile

**Fig. 1:**
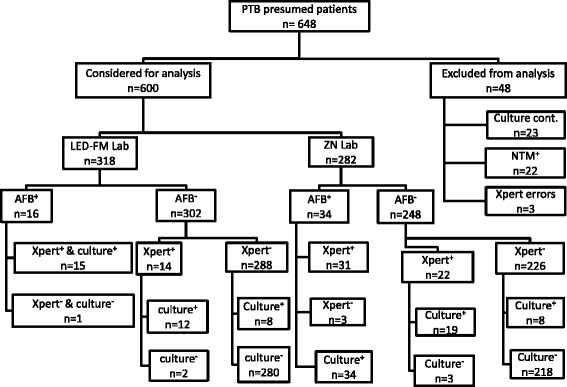
Flow chart showing series of participants’ recruitment and the outcome of different tuberculosis testing methods used. +: Positive, **-**: Negative, AFB: Acid Fast Bacilli, PTB: Pulmonary tuberculosis, ZN Lab: Ziehl Nelsen health facility laboratories-, LED-FM Lab: Light emitting diode-fluorescence microscopy using health facility laboratories, NTM: Non-tuberculous Mycobacteria, cont.: contaminated

Among the 648 patients enrolled, 48 were excluded for analysis due to incomplete results (23; 3.5 % contaminated cultures, 22; 3.4 % cultures positive for NTMs and 3; 0.5 % invalid Xpert results). Of the 600 included, 372 (62 %) were male and median age was 37 years (inter-quartile range 28–50). A total of 390 (65.0 %) and 210 (35.0 %) participants were from PLs and ILs respectively of whom, 318 (53 %) were from laboratories performing ZN and 282 (47 %) from laboratories performing LED-FM microscopy, Fig. 1.

The prevalence of HIV in this study population was 162 (27.0 %). The smear positivity rates were 12.0 % and 5.0 % among ZN and LED-FM laboratories respectively, whereas the positivity rate for Xpert was 13.7 % of whom seven (1.2 %) had rifampicin resistant *M. tuberculosis*. A total of 96 (16.0 %) participants had culture confirmed TB of whom 28 (29.2 %) were HIV-infected, Table 1.

**Table 1:** Participant characteristics (*n* = 600)

Characteristic	Frequency	Percentage
Sex		
Male	372	62.0
Female	228	38.0
Age category		
1 (15–35)	287	47.8
2 (36–55)	202	33.7
3 (≥56)	111	18.5
HIV status		
Positive	162	27.0
Negative	438	73.0
Mycobacterial testing results		
ZN smear positive	34	12.1
LED-FM smear positive	16	5.0
Xpert positive	82	13.7
Culture (MGIT and/or LJ) positive	96	16.0

*MGIT* mycobacterial growth indicator tube; *LJ* Lowenstein Jensen; *LED-FM* light emitting diode fluorescence microscopy; *ZN* Ziehl Nelsen Xpert: Xpert MTB/RIF test; *HIV* human immunodeficiency virus

### Senstivity of smear methods and Xpert assay

Among 96 culture positive for *M. tuberculosis* by MGIT and/or LJ method, 47 (49.0 %) were smear negative of which 20 (42.6 %) were tested with LED-FM and 27 (57.4 %) with ZN. Among 82 patients found MTB positive with the Xpert assay, 36 (43.9 %) were smear microscopy negative of whom 13 (36.1 %) were HIV-infected. In regard to rifampicin resistance, seven rifampicin resistant MTB cases were detected by Xpert, among which only two were smear positive. The overall sensitivity of Xpert was 80.2 %, 95%CI (70.8–87.6). Xpert at PLs had a sensitivity of 77.6 %, 95%CI (66.6–86.4) as compared to 90 %, 95%CI (68.3–98.8) among ILs the overall sensitivity of smear microscopy was 51.0 %, 95 % CI (40.6–61.4 %). Overall sensitivity for smear microscopy among PLs was 48.7 %, 95 % CI (37.0–60.4) whereas for ILs was 60.0 %, 95%CI (36.1–80.9). The overall sensitivity of smear microscopy was 39.3 %, 95%CI (21.5–59.4) and 55.9 %, 95%CI (43.3–67.9) among HIV-positive and HIV-negative TB patients respectively. By smear microscopy method, smear by ZN at PLs had sensitivity of 55.1 %, 95 % CI (40.2–69.3) as compared to LED-FM 37.7 %, 95%CI (19.4–57.6). For ILs, the sensitivity for ZN smear microscopy was 58.3 %, 95%CI (27.7–84.8) as compared to LED-FM 62.5 %, 95%CI (24.5–59.4), Table 2. The overall specificity of smear microscopy 99.8 % 95 % C.I (99.4–100 %). The overall sensitivity of smear microscopy was 55.7 %, 95 % C.I (42.4–68.5–63.2 %) and 42.9 % (26.3–60.6 %) among ZN and LED-FM using laboratories respectively, Table 2.

**Table 2:** Yield of smear microscopy versus Xpert among culture confirmed tuberculosis patients (*n* =96)

		Xpert		Sensitivity of SM % (95 %, CI)	Incremental sensitivity of Xpert % (95 %,CI)
		Positive	Negative		
Overall SM at all HF *n* = 96	positive	46	3	51.0 (40.6–61.4)	32.3 (23.1–42.6)
	negative	31	16		
SM in all HIV positive *n* = 28	positive	10	1	39.3 (21.5–59.4)	35.7 (18.6–55.9)
	negative	10	7		
SM in all HIV negative *n* = 68	positive	36	2	55.9 (43.3–67.9)	30.9 (20.2–43.3)
	negative	21	9		
SM at all PL *n* = 76	positive	34	3	48.7 (37.0–60.4)	32.9 (22.5–44.6)
	negative	25	14		
SM at all IL *n* = 20	positive	12	0	60.0 (36.1–80.9)	30.0 (11.9–54.3)
	negative	6	2		
SM at ZN PL *n* = 49	positive	24	3	55.1 (40.2–69.3)	32.7 (19.9–47.5)
	negative	16	6		
SM at LED-FM PL *n* = 27	positive	10	0	37.0 (19.4–57.6)	33.3 (16.5–54.0)
	negative	9	8		
SM at ZN IL *n* = 12	positive	7	0	58.3 (27.7–84.8)	25.0 (5.5–57.2)
	negative	3	2		
SM at LED-FM IL *n* = 8	positive	5	0	62.5 (24.5–91.5)	37.5 (8.5–75.5)
	negative	3	0		

*LED-FM* light emitting diode fluorescence microscopy; *ZN* Ziehl Nelsen Xpert: Xpert MTB/RIF test; *HIV* human immunodeficiency virus; *CI* confidence interval; *SM* smear microscopy; *IL* intermediate laboratories; *PL* peripheral laboratories; *SM* smear microscopy

### Incremental senstivity of Xpert assay over the smear microscopy results

The overall incremental sensitivity (IS) of Xpert over smear microscopy at all HF was 32.3 %, 95 % CI (23.1–42.6 %). The overall IS of Xpert for either smear microscopy was 35.7 %, 95%CI (18.6–55.9) and 30.9 %, 95%CI (20.2–43.3) among HIV-positive and HIV-negative individuals respectively. The IS of Xpert among PLs using ZN was 32.7 %, 95 % CI (19.9–47.5); whereas for PLs using LED-FM was 33.3, 95%CI (16.5–54.0). Among ILs using ZN, the IS of Xpert was 25.0 %, 95%CI (5.5–57.2) as compared to ILs using LED-FM 37.5 %, 95%CI (8.5–75.5), Table 2.

## Discussion

In this cross-sectional study aimed at assessing the accuracy of smear microscopy and the incremental sensitivity of Xpert in presumptive pulmonary tuberculosis patients at tuberculosis laboratories in Rwanda, we document low sensitivity of sputum smear microscopy in tuberculosis diagnostic laboratories, particularly in peripheral laboratories. The added value of Xpert was particularly important among HIV-infected patients and for detection of drug-resistant cases. We further confirm a significant gain from Xpert when used as an initial diagnostic test at health facility laboratories. For both health facility levels, the sensitivity of Xpert was significantly higher than either smear microscopy methods. As expected, the sensitivities of both smear methods including Xpert was higher among HIV-negative participants. Replacing ZN smear microscopy with LED-FM did not increase the detection of TB at both health facility levels. The incremental detection of Xpert from both smear methods was significantly higher among PLs but not at ILs.

The sensitivity found in this study was however in the range of findings from several studies where the sensitivities of conventional ZN microscopy ranged from 32 to 94 % and the sensitivities of fluorescence microscopy ranged from 52 to 97 %, with the fluorescent method being on average 10 % more sensitive than light microscopy [5]. In contrast to earlier findings, however, the current study did not find the sensitivity of LED-FM to be statistically different from ZN (*p* = 0.371). These results differ from several previous studies where LED-FM increases an average of 10 % of sensitivity over the conventional ZN technique [6–10]. Other studies have shown equal sensitivity or low specificity of LED-FM compared to conventional ZN technique [9, 11–13]. In these studies, readers had no previous experience with fluorescence microscopy, which is the most likely explanation for sensitivity differences compared with other studies and indicating the importance of adapting training intensity according to the level of operator proficiency. Our findings showed that smear microscopy performed better at intermediate laboratories compared to peripheral; the sensitivity for LED-FM 62.5 % vs. 37.0 %, *p* = 0.023 but not for ZN 58.3 % vs.55.1 %; *p* = 0.265. This may either be explained by the fact that LED-FM was implemented at IL prior to peripheral and therefore technologists acquired experience earlier compared to those at IL or due to small sample size at the ILs.

The sensitivity of sputum smear microscopy in HIV-infected participants was found to be low and are in agreement with findings of previous studies, where it ranges from 30 to 48 % [14–16]. The poor performance of sputum smear microscopy in HIV patients can be explained in part by the fact that pulmonary tuberculosis in these patients presents with paucibacillary TB and lack cavitation [16].

The overall prevalence of smear negative pulmonary tuberculosis using either smear method in HIV-infected PTB presumptive participants was found to be high and are in line with those of previous studies [17, 18]. The level of immunosuppression among HIV-infected patients affects significantly the results of the sputum smear; less severely immunocompromised HIV-positive patients tend to have classic cavitary tuberculosis with smear-positive results; as the level of immunocompromised increases with advancing HIV disease, atypical pulmonary features predominate and smear examinations prove less sensitive [17].

Although, the overall sensitivity of Xpert for the detection of *M. tuberculosis* was slightly lower, its specificity was consistent with those of previous studies in a Cochrane review even when stratified by HIV status [4]. The insignificant incremental sensitivity of Xpert test over smear microscopy at ILs is likely to be more explained by the small sample size we had at these facilities.

The significant incremental sensitivity of Xpert from either smear method at PLs supports the WHO recommendation for using Xpert as an initial test for TB diagnosis [19]. Although the cost per test and compulsory required maintenance of Xpert machine (annual calibration, replacement of modules, good and constant power supply) may be not affordable by many poor resource settings given the limited heath budgets [20], the savings from increased case detection and timely initiation of treatment due to early diagnosis, may be more cost effective in terms of supplies savings as well as patient savings from repeat facility visits. In addition, early diagnosis may reduce the risk of TB transmission. It is worth noting that the effectiveness of Xpert testing is likely to depend on utilization as the test tends to be less effective in low workload settings [21] as the low numbers of patients tested at IL could have affected the strength of obtained significance measure.

Our study had some limitations; the level of immunosuppression for HIV positive PTB presumptive participants was not measured (CD4); this could lead to poor classification and consequently low differences in terms of smear sensitivity among HIV-infected participants. Secondary, the low samples size obtained from ILs which probably masks the obvious significant incremental detection of Xpert; among the 600 participants of this study, only 210 (35 %) participants and 20 (20.8 %) pulmonary tuberculosis confirmed cases were from ILs. The few numbers of HIV-infected patients in this study could not allow for meaningful comparison of incremental sensitivity by HIV-status, however, significant Xpert IS among HIV-infected patients was previously documented [22]. Lastly, excluding samples which became contaminated or positive for an NTM from the analysis may have led to an under-estimation of the sensitivity of Xpert and smear microscopy, as tuberculosis cannot be definitively excluded for these patients. TB-NTM coinfection has been reported, but is supposed to be a relatively rare clinical entity in Rwanda. However, excluding these participants may have over-estimated the specificity of microscopy, as smear-positive NTM infections were not taken into account in the analysis, while the specificity of Xpert would probably have been much less affected by the presence of NTM.

## Conclusions

The findings from this study revealed a low detection rate of both LED-FM and ZN smear microscopy at health facility tuberculosis diagnostic laboratories in Rwanda. This study revealed a low sensitivity of LED-FM smear microscopy compared to ZN-microscopy among PLs whereas for ILs, the senstivity of LED-FM was higher than that of ZN microscopy, indicating differences in skills requirements among microscopy methods. This study revealed a signicant incremental detection gained from Xpert. Hence, the data from this study strongly support the conditional recommendation of WHO for Xpert; where the Xpert may be used as initial diagnostic test in adults and children presumed to have TB. Nevertheless, other studies of cost-effectiveness and feasibility of the proposed strategy at large scale are necessary.

## Abbreviations

AFB: acid fast bacilli
BD: Becton Dickinson Company
FM: fluorescence microscopy
HIV: human immunodeficiency virus
ILs: intermediate laboratories
LED-FM: light emitting diode fluorescence microscopy
LJ: Löwenstein-Jensen
MDR-TB: multi-drug resistant tuberculosis
MGIT: mycobacterium growth indicator tube
MTB: *mycobacterium tuberculosis* complex
NRL: National Reference Laboratory
NTM: Non Tuberculosis Mycobacterium
PLs: peripheral laboratories
PTB: pulmonary tuberculosis
RIF: rifampicin
TB: tuberculosis
WHO: World Health Organization
ZN: Ziehl Neelsen staining

## References

[1] Drobniewski F, Balabanova Y, Nikolayevsky V, Ruddy M, Kuznetzov S, Zakharova S, Melentyev A, Fedorin I (2005). Drug-resistant tuberculosis, clinical virulence, and the dominance of the Beijing strain family in Russia. JAMA.

[2] Islam MR, Khatun R, Uddin MK, Khan MS, Rahman MT, Ahmed T, Banu S (2013). Yield of two consecutive sputum specimens for the effective diagnosis of pulmonary tuberculosis. PLoS One.

[3] Chaidir L, Parwati I, Annisa J, Muhsinin S, Meilana I, Alisjahbana B, van Crevel R (2013). Implementation of LED fluorescence microscopy for diagnosis of pulmonary and HIV-associated tuberculosis in a hospital setting in Indonesia. PLoS One.

[4] Steingart KR, Sohn H, Schiller I, Kloda LA, Boehme CC, Pai M, Dendukuri N (2013). Xpert(R) MTB/RIF assay for pulmonary tuberculosis and rifampicin resistance in adults. Cochrane Database Syst Rev.

[5] Steingart KR, Henry M, Ng V, Hopewell PC, Ramsay A, Cunningham J, Urbanczik R, Perkins M, Aziz MA, Pai M (2006). Fluorescence versus conventional sputum smear microscopy for tuberculosis: a systematic review. Lancet Infect Dis.

[6] Marais BJ, Brittle W, Painczyk K, Hesseling AC, Beyers N, Wasserman E, van Soolingen D, Warren RM (2008). Use of light-emitting diode fluorescence microscopy to detect acid-fast bacilli in sputum. Clin Infect Dis.

[7] Trusov A, Bumgarner R, Valijev R, Chestnova R, Talevski S, Vragoterova C, Neeley ES (2009). Comparison of Lumin LED fluorescent attachment, fluorescent microscopy and Ziehl-Neelsen for AFB diagnosis. Inte J Tuberc Lung Disease.

[8] Albert H, Manabe Y, Lukyamuzi G, Ademun P, Mukkada S, Nyesiga B, Joloba M, Paramasivan CN, Perkins MD (2010). Performance of three LED-based fluorescence microscopy systems for detection of tuberculosis in Uganda. PLoS One.

[9] Cuevas LE, Al-Sonboli N, Lawson L, Yassin MA, Arbide I, Al-Aghbari N, Sherchand JB, Al-Absi A, Emenyonu EN, Merid Y (2011). LED fluorescence microscopy for the diagnosis of pulmonary tuberculosis: a multi-country cross-sectional evaluation. PLoS Med.

[10] Lehman LG, Ngapmen Yamadji AL, Ngo Sack F, Bilong Bilong CF (2010). The CyScope(R) fluorescence microscope, a reliable tool for tuberculosis diagnosis in resource-limited settings. AmJTrop Med Hyg.

[11] Bonnet M, Gagnidze L, Githui W, Guerin PJ, Bonte L, Varaine F, Ramsay A (2011). Performance of LED-based fluorescence microscopy to diagnose tuberculosis in a peripheral health centre in Nairobi. PLoS One.

[12] Shenai S, Minion J, Vadwai V, Tipnis T, Shetty S, Salvi A, Udwadia Z, Pai M, Rodrigues C (2011). Evaluation of light emitting diode-based fluorescence microscopy for the detection of mycobacteria in a tuberculosis-endemic region. Int J Tuberc Lung Disease.

[13] Turnbull ER, Kaunda K, Harris JB, Kapata N, Muvwimi MW, Kruuner A, Henostroza G, Reid SE (2011). An evaluation of the performance and acceptability of three LED fluorescent microscopes in Zambia: lessons learnt for scale-up. PLoS One.

[14] Whitelaw A, Peter J, Sohn H, Viljoen D, Theron G, Badri M, Davids V, Pai M, Dheda K (2011). Comparative cost and performance of light-emitting diode microscopy in HIV-tuberculosis-co-infected patients. Eur Respir J.

[15] Albert H, Nakiyingi L, Sempa J, Mbabazi O, Mukkada S, Nyesiga B, Perkins MD, Manabe YC (2013). Operational Implementation of LED Fluorescence Microscopy in Screening Tuberculosis Suspects in an Urban HIV Clinic in Uganda. PLoS One.

[16] Swaminathan S, Padmapriyadarsini C, Narendran G (2010). HIV-associated tuberculosis: clinical update. Clin Infect Dis.

[17] Colebunders R, Bastian I (2000). A review of the diagnosis and treatment of smear-negative pulmonary tuberculosis. Int J Tuberc Lung Dis.

[18] Elliott AM, Hayes RJ, Halwiindi B, Luo N, Tembo G, Pobee JO, Nunn PP, McAdam KP (1993). The impact of HIV on infectiousness of pulmonary tuberculosis: a community study in Zambia. AIDS (London, England).

[19] World Health Organization; Tuberculosis diagnostics, Xpert MTB/RIF Test. WHO Recommendations October 2013; Available: http://www.who.int/tb/publications/Xpert_factsheet.pdf?ua=1. Accessed 1 Aug 2014.

[20] van’t Hoog AH, Cobelens F, Vassall A, van Kampen S, Dorman SE, Alland D, Ellner J (2013). Optimal triage test characteristics to improve the cost-effectiveness of the Xpert MTB/RIF assay for TB diagnosis: a decision analysis. PLoS One.

[21] World Health Organization, 2013. Xpert MTB/RIF Implementation Manual: Technical and Operational ‘How-To’; Practical Considerations. Available; http://www.ncbi.nlm.nih.gov/books/NBK254329/. Accessed 22 Feb 2016

[22] Ssengooba W, Nakiyingi L, Armstrong DT, Cobelens FG, Alland D, Manabe YC, Dorman SE, Ellner JJ, Joloba ML (2014). Clinical utility of a novel molecular assay in various combination strategies with existing methods for diagnosis of HIV-related tuberculosis in Uganda. PLoS One.

